# Test‐time adaptation‐driven patient‐specific auto‐segmentation for CBCT‐guided online adaptive radiotherapy in cervical cancer

**DOI:** 10.1002/acm2.70609

**Published:** 2026-05-14

**Authors:** Zhiqun Wang, Wei Zhang, Xiangyin Meng, Yongguang Liang, Tingting Dong, Jie Zhang, Nan Liu, Lang Yu, Bo Yang, Qichao Zhou, Jie Qiu

**Affiliations:** ^1^ Department of Radiation Oncology Chinese Academy of Medical Sciences, Peking Union Medical College Beijing China; ^2^ Manteia Technologies Co., Ltd. Xiamen Fujian China

**Keywords:** cone‐beam computed tomography (CBCT), patient‐specific segmentation, test‐time adaptation (TTA)

## Abstract

**Background:**

Online adaptive radiotherapy using cone beam computed tomography (CBCT) of the pelvis can account for anatomical variations during cervical cancer treatment. However, the limitations of CBCT and existing auto‐segmentation methods, such as deformable image registration (DIR), hinder contouring and dosimetric accuracy.

**Purpose:**

We propose a test‐time adaptation (TTA)‐driven patient‐specific segmentation framework to enhance the accuracy and workflow efficiency of CBCT‐guided online adaptive radiotherapy in cervical cancer.

**Methods:**

Our retrospective analysis included 20 patients with cervical/endometrial cancer (530 CBCT scans) treated on the Varian Ethos platform. A patient‐specific UNet variant pre‐trained on the CTPelvic1K dataset using Bootstrap Your Own Latent (BYOL) contrastive learning was fine‐tuned on individual reference CT scans. During daily adaptation, the model incorporated TTA with segmentation (cross entropy + Dice loss) and consistency objectives, leveraging prior CBCT/CT images to constrain anatomical variations. Performance was evaluated using the Dice similarity coefficient (DSC), 95th percentile Hausdorff Distance (HD95), and contouring time reduction compared to DIR‐based methods.

**Results:**

The proposed method significantly outperformed DIR, achieving higher DSC (e.g., postoperative clinical target volume (CTV) of the vaginal cuff and upper vaginal region: 0.89 vs. 0.73; definitive CTV of the pelvic lymphatic drainage area: 0.83 vs. 0.71) and lower HD95 (e.g., rectum: 24.9 vs. 30.7 mm) values. TTA further improved the accuracy, particularly for the bowel (DSC: 0.81 vs. 0.76) and target volumes. Clinically, the proposed method reduced the contouring time by 250 s (postoperative) and 230 s (definitive) per fraction (*p* < 0.01), with reduced time variability. Structures with clear boundaries required one or two fractions for training, whereas motion‐prone organs required four or five fractions.

**Conclusions:**

This study presents the first retrospective demonstration of TTA‐driven patient‐specific segmentation for pelvic CBCT‐guided online adaptive radiotherapy. The proposed framework enhances segmentation accuracy, workflow efficiency, and temporal consistency by addressing the inherent challenges and data scarcity of CBCT through adaptive learning.

## 1 INTRODUCTION

Online adaptive radiation therapy is a transformative approach that dynamically modifies radiotherapy plans during treatment to account for anatomical variations and improve targeting accuracy and treatment efficacy. Recent advances in imaging technologies, including cone beam computed tomography (CBCT), computed tomography (CT), and magnetic resonance imaging (MRI), as well as developments in automation, have facilitated rapid online image acquisition, treatment plan modification, and radiation therapy.^1^, ^2^ CBCT is a crucial imaging tool for adaptive radiotherapy owing to its accessibility and cost‐effectiveness when integrated with modern linear accelerators.^3^ However, although CBCT enables rapid volumetric imaging for daily treatment guidance, it remains limited by poor soft‐tissue contrast and image noise, which has prompted the development of advanced reconstruction algorithms and artificial intelligence (AI)‐based enhancement techniques.^4^, ^5^

Automatic segmentation methods in online adaptive radiation therapy can be divided into two main categories: deformable image registration (DIR)‐based methods and AI‐based approaches. The former generates contours by propagating manually delineated structures from reference images to new scans using deformable registration algorithms,^6^ demonstrating versatility across different anatomical sites and typically achieving moderate accuracy with Dice similarity coefficients (DSC) of approximately 0.7.^7^ However, deformable registration methods exhibit substantial sensitivity to anatomical variations in pelvic malignancies, where changes in bladder and rectal filling states can induce substantial discrepancies in the target volume and organs at risk (OARs) between reference and target images.^7^ Such variations can increase the contouring time by 30%–45% and may compromise dosimetric accuracy.^8^

In contrast, AI‐based approaches, particularly deep learning models, exhibit superior performance by learning complex feature representations from large annotated datasets, frequently achieving DSCs > 0.9 or 0.84–0.89 for pelvic OAR segmentation.^9^, ^10^ AI‐powered auto‐segmentation systems have also shown promise for addressing challenges^11^ related to anatomical variations, reducing inter‐observer variability by up to 60% and improving contouring efficiency by 40%–50% compared to manual methods.^12^, ^13^ Moreover, the speed and accuracy of AI‐driven CBCT‐guided online adaptive radiotherapy^14^ now meet clinical requirements, enabling CBCT imaging to transcend its traditional role as a mere image‐guidance tool and play a pivotal role in managing anatomical variations during treatment and addressing the growing precision demands of modern radiotherapy.

However, AI‐based methods remain constrained by their dependence on large, high‐quality training datasets and computational resources, showing performance degradation for rare anatomical presentations that are not well represented in training data,^8^ as well as challenges in model interpretability and clinical workflow integration.^9^ A notable advance in this field is the Ethos adaptive radiation therapy system (Varian Medical Systems, Palo Alto, California, USA), which integrates dose delivery and iterative CBCT imaging into a unified platform. The Ethos workflow employs AI‐generated “influencer” structures to expedite the initial setup. Nevertheless, in clinical practice, these structures typically require meticulous manual adjustments to ensure their accuracy. Although this customization step is critical for precision, it inevitably prolongs the overall workflow.

Therefore, in this retrospective study, we propose a test‐time adaptation (TTA)‐driven patient‐specific auto‐segmentation framework to enhance the accuracy and contouring workflow efficiency of CBCT‐guided adaptive radiotherapy for cervical cancer. Our analysis is based on archived clinical data from patients treated on the Varian Ethos platform.

## 2 METHODS

### 2.1 Patients

This retrospective study was approved by the Ethics Committee of our center. We analyzed an archived dataset of 20 patients with endometrial or cervical cancer who underwent daily CBCT‐guided online adaptive radiotherapy on a Varian Ethos platform version 1.0 (Varian Medical Systems) between October 2022 and March 2023. All data, including reference CT scans, daily CBCT images, and corresponding contours, were retrieved from our institutional archive for offline analysis. The proposed TTA framework and all contour evaluations were performed in a research environment and were not part of the real‐time clinical treatment workflow. The cohort included 10 patients who received postoperative radiotherapy (following hysterectomy for cervical or endometrial cancer) and 10 patients who were treated with definitive radiotherapy for cervical cancer.

All reference CT scans were acquired using a spectral CT scanner (Revolution, GE Healthcare) with the following parameters: tube voltage, 140 kVp; tube current, 324 mA; rotation time, 0.8 s; and slice thickness, 5 mm, in accordance with our institutional protocol for pelvic radiotherapy planning. For each treatment session, CBCT images were obtained using an iterative CBCT system (Varian Ethos) at 125 kVp, 1080 mAs, and a reconstructed slice thickness of 2 mm.

Target volumes and OARs were delineated on both reference CT and CBCT images. In the postoperative group, the clinical target volume (CTV) was subdivided into the following: CTV‐V (vaginal cuff and upper vaginal region) and CTV‐N (pelvic lymphatic drainage area). These patients were prescribed 1.8 Gy per fraction (session) for more than 25 fractions. For the definitive radiotherapy group, the CTV included: CTV‐C (cervical region), CTV‐U (uterine body), and CTV‐N. These patients received 1.8 Gy per fraction over 28 fractions. The delineated OARs included the bladder, rectum, small bowel (Bowel denotes small bowel throughout the text), bilateral femoral heads, spinal cord, and bone marrow. The final dataset consisted of reference CT scans from all 20 patients and 530 CBCT images with their corresponding contours.

### 2.2 Patient‐specific pre‐trained model building

We utilized extensive and diverse pelvic CT scans from the CTPelvic1K dataset to develop our pre‐trained model. The CTPelvic1K dataset was selected due to its large‐scale, multi‐center collection of pelvic CT scans with expert‐annotated OARs, which provides robust anatomical diversity for pre‐training deep segmentation models in pelvic radiotherapy.^15^ Notably, no manual contours were used from the CTPelvic1K dataset, which only provided raw CT images for pre‐training. We adopted Bootstrap Your Own Latent (BYOL)^16^ for unsupervised pre‐training because it achieves competitive representation learning without negative samples, which is advantageous when working with limited labeled medical data and avoids reliance on large batch sizes. The model architecture is a sophisticated variant of UNet that incorporates multiple residual modules to enhance feature extraction and representation. This cutting‐edge design, illustrated in Figure 1, was tailored to capture intricate details and contextual nuances from CT imagery. To further bolster the model capabilities, we leveraged the BYOL contrastive learning method for unsupervised pre‐training of the encoder. This innovative approach allowed us to derive rich and meaningful representations from unlabeled data, which served as the foundation for initializing the segmentation network tailored to each individual patient. Subsequently, we employed a patient‐specific training approach, utilizing only the reference CT scan and corresponding contours for each patient as the input data. This ensured that each patient received a highly personalized segmentation model that was fine‐tuned to the unique anatomical characteristics and variations present in their CT images. Considering the transition of the pre‐trained model from CT images to its eventual application on CBCT images, we incorporated rigorous image enhancement techniques during the segmentation model training. These enhancements encompassed a range of strategies, including spatial and intensity transformations and the simulation of various artifacts, to ensure model versatility and robust performance across different imaging modalities.

**FIGURE 1:**
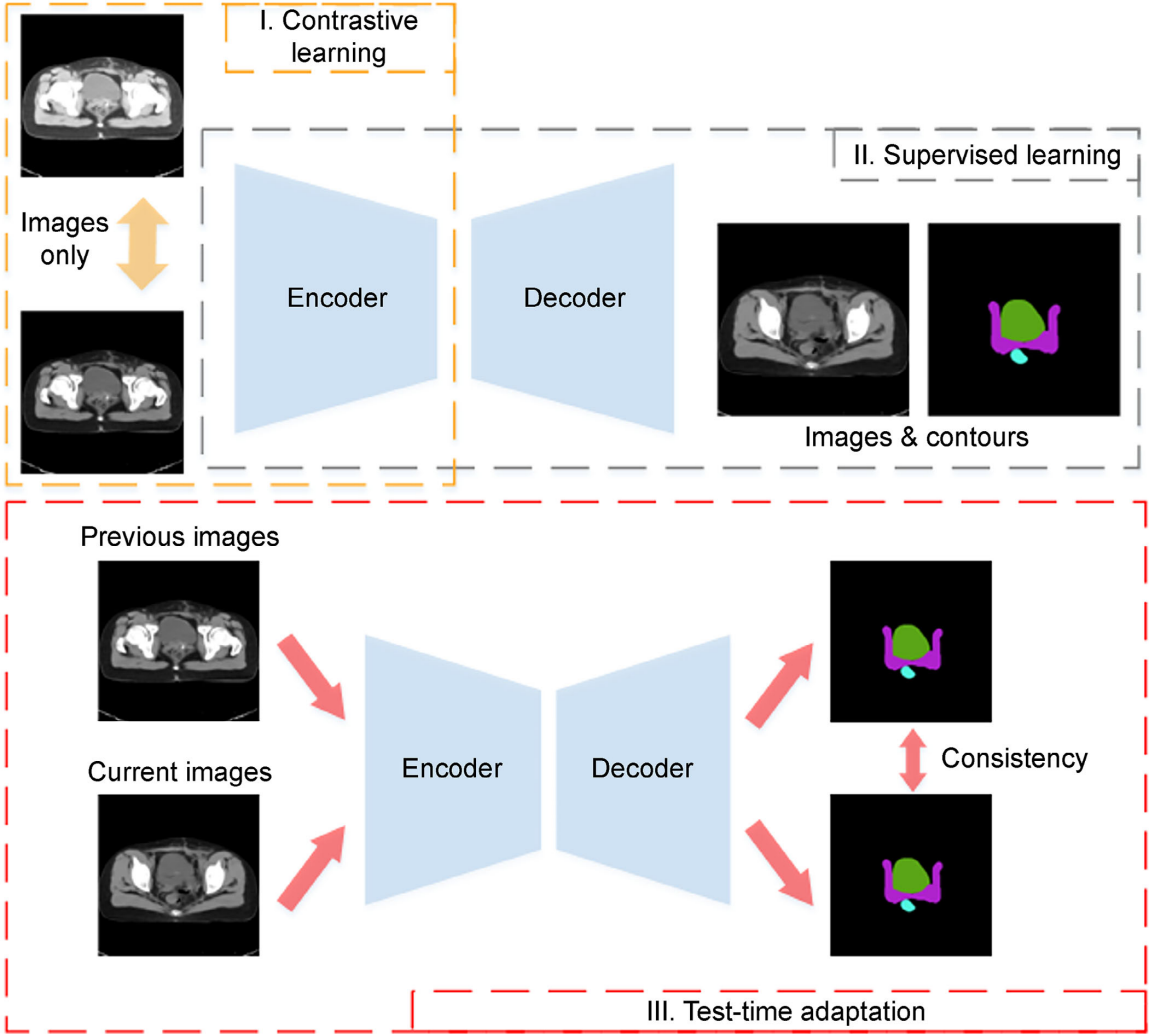
Construction of the patient‐specific segmentation model and test‐time adaptation strategy.

### 2.3 Consistency‐based TTA

We performed TTA of the model before deploying it to daily CBCT images. This crucial step relies primarily on maintaining consistency between the data and model variations. Given the expectation that patient anatomy and target volume would undergo only minor changes between consecutive treatments, we imposed explicit constraints during model adaptation. To this end, we utilized the current CBCT image for each patient along with their previous CBCT images (or the reference CT prior to the initial treatment) and the corresponding contours; these serve as the inputs for the adaptation process. The model was designed with two distinct objectives: a segmentation objective $\theta_{seg}$ and a consistency objective $\theta_{cons}$. The former focuses solely on the previous images $x_{prev}$ and contours $y_{prev}$, whereas the latter enforces a specific range of variation between the current images $x_{cur}$ and previous images. Specifically, $x_{prev}$ is input to the patient‐specific segmentation model that has been pre‐trained to obtain the prediction $p_{prev}$. The segmentation objective $\theta_{seg}$ consists of cross entropy and Dice loss, expressed as

$$\begin{array}{cc} \theta_{seg} & =-\left[y_{prev}\cdot \log\left(p_{prev}\right)+\left(1-y_{prev}\right)\cdot \log\left(1-p_{prev}\right)\right]\\ & \;+1-\frac{2\cdot p_{prev}\cap y_{prev}}{\left|p_{prev}\right|+\left|y_{prev}\right|} \end{array}$$

To ensure that TTA preserves the original patient features while adapting to the current images, we incorporated a consistency objective function $\theta_{cons}$. If we let $p_{cur}$ be the model prediction for the current image, then

$$\theta_{cons}=-\log\left(\frac{exp\left(p_{cur}\right)}{exp\left(p_{cur}\right)+exp\left(p_{prev}\right)}\right)$$

The total adaptation loss is formulated as $\theta_{total}=\theta_{seg}+\lambda \cdot \theta_{cons}$, where λ=0.5. This value was selected empirically based on validation experiments on five hold‐out patients to balance segmentation fidelity and temporal smoothness. Prior to TTA, the current CBCT image was aligned to the previous image $x_{prev}$ (or the reference CT for the first fraction) using rigid registration only, as implemented in the Ethos platform. We deliberately avoided deformable registration due to its known sensitivity to large anatomical changes in the pelvis (e.g., bladder/rectal filling variations), which can introduce erroneous deformations and propagate contouring errors. Instead, our consistency objective $\theta_{cons}$ is designed to encourage the model to adapt to genuine anatomical shifts while preserving learned structural priors from the patient‐specific initialization—thereby reducing reliance on potentially unreliable DIR.

### 2.4 Evaluation metrics

To evaluate the effectiveness of the proposed method, we selected DSC and 95th percentile Hausdorff Distance (HD95) as primary evaluation metrics, following established guidelines for medical image segmentation validation.^17^ DSC quantifies volumetric overlap, while HD95 captures boundary localization accuracy—both are critical for radiotherapy contouring.

### 2.5 Comparison of contouring time differences

To ensure clinical relevance, two senior radiation oncologists performed independent blinded evaluations and modifications of all segmentation results. Radiation oncologists were instructed to adjust any contour deemed clinically inaccurate using standard tools in the Ethos system (e.g., brush editing, slice‐by‐slice correction, or propagation with manual override). No constraints were placed on the extent of modification, but all edits were logged automatically by the system for time tracking. The time required for each evaluation and modification session was documented in detail. In clinical practice, evaluation and modifications were conducted using the Ethos online contouring system, whereas model‐generated structures were processed using Eclipse version 15.5 contouring tools. As both experts were highly familiar with both systems, the differences in proficiency between the two platforms were considered negligible. Time records and analyses were used to evaluate the overall clinical feasibility and time‐efficiency of the proposed method.

### 2.6 Statistic analysis

All analyses were performed using Python 3.9 with SciPy v1.10.0. Given the limited size of the available dataset and the paired nature of the comparisons, a two‐tailed Wilcoxon signed‐rank test was selected as the statistical method for validation.

## 3 RESULTS

To evaluate the clinical efficacy of our method, we compared the structural modification time for two clinical scenarios for cervical cancer, postoperative radiotherapy​ and ​definitive radiotherapy, using conventional workflows and our proposed approach, as shown in Figure 2. In the conventional clinical workflow, the time required from target volume generation to finalization (including contouring modifications and approval by radiation oncologists) was 665.3 ± 117.9 s for postoperative radiotherapy and 626.3 ± 222.1 s for definitive radiotherapy. In contrast, when using our method to generate initial target volumes followed by physician modifications and approval, the time was significantly reduced to 416.3 ± 43.7 s and 398.0 ± 47.9 s, respectively, achieving overall time savings of approximately 250 s and 230 s (*p* < 0.01). Notably, the variability in contouring time was markedly reduced. This reduction in temporal fluctuations is particularly critical for online adaptive radiotherapy​ because it enables more stable intra‐fractional variations to guide margin expansion for target volumes.

**FIGURE 2:**
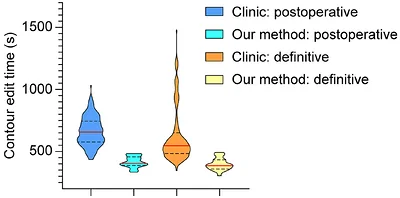
Contouring modification time for patients receiving postoperative and definitive radiotherapy under clinical workflows versus our proposed method.

To evaluate the effectiveness of the proposed method, we conducted a comparative analysis with common DIR techniques used in adaptive radiotherapy. The results are summarized in Tables 1 and 2. Overall, the proposed method consistently outperforms DIR in terms of accuracy across all regions of interest, regardless of whether TTA was applied. Notably, when TTA was introduced, our method exhibited further improvements over the non‐TTA version, particularly in the target volumes and bowel regions. Moreover, the proposed method achieved lower standard deviations in both DSC and HD95 values than traditional DIR techniques, indicating superior precision and consistency.

**TABLE 1:** DICE values for all test cases.

	Deform reg	Proposed	Proposed TTA	*p*‐value proposed vs. proposed TTA
Postoperative				
CTV‐N	0.70 ± 0.14	0.80 ± 0.08	**0.84 ± 0.04**	**0.002**
CTV‐V	0.73 ± 0.12	0.86 ± 0.04	**0.89 ± 0.03**	**≤0.001**
Definitive				
CTV‐N	0.71 ± 0.14	0.79 ± 0.09	0.83 ± 0.07	0.015
CTV‐V	0.74 ± 0.11	0.86 ± 0.05	**0.88 ± 0.06**	**≤0.001**
CTV‐U	0.66 ± 0.15	0.76 ± 0.10	0.79 ± 0.08	0.10
Bladder	0.83 ± 0.09	0.92 ± 0.05	0.93 ± 0.04	0.27
Rectum	0.70 ± 0.15	0.82 ± 0.05	0.84 ± 0.05	0.048
Femur head	0.86 ± 0.08	0.93 ± 0.02	0.92 ± 0.02	0.014
Spinal cord	0.72 ± 0.13	0.89 ± 0.03	0.88 ± 0.03	0.099
Bowel	0.69 ± 0.14	0.76 ± 0.05	**0.81 ± 0.05**	**≤0.001**
Bone marrow	0.79 ± 0.08	0.89 ± 0.02	0.90 ± 0.02	0.014

*Note*: Underlined values show a significant advantage (*p* < 0.01, proposed vs. proposed TTA). Deform reg: deformation registration. Proposed: patient‐specific model. Proposed TTA: patient‐specific model with test‐time adaptation.

**TABLE 2:** HD95 values for all test cases.

	Deform reg	Proposed	Proposed TTA	*p*‐value proposed vs. proposed TTA
Postoperative				
CTV‐N	17.8 ± 7.2	15.7 ± 5.7	13.2 ± 4.2	0.014
CTV‐V	16.2 ± 5.8	15.6 ± 6.3	12.5 ± 5.5	0.011
Definitive				
CTV‐N	19.6 ± 4.1	16.9 ± 6.8	14.6 ± 5.1	0.059
CTV‐V	16.4 ± 3.2	15.7 ± 5.4	13.9 ± 4.3	0.068
CTV‐U	16.7 ± 2.8	15.8 ± 5.6	14.7 ± 3.9	0.257
Bladder	14.8 ± 2.7	13.9 ± 2.2	**10.9 ± 1.8**	**≤0.001**
Rectum	30.7 ± 5.2	27.5 ± 4.7	**24.9 ± 4.1**	**0.004**
Femur Head	8.2 ± 1.8	7.6 ± 2.9	6.3 ± 2.1	0.012
Spinal Cord	6.7 ± 2.6	4.3 ± 1.1	4.2 ± 0.9	0.620
Bowel	60.8 ± 22.3	48.3 ± 18.8	40.3 ± 17.3	0.029
Bone Marrow	20.2 ± 8.3	18.6 ± 6.6	16.5 ± 5.8	0.094

*Note*: Underlined values show a significant advantage (*p* < 0.01, proposed vs. proposed TTA). Deform reg: deformation registration. Proposed: patient‐specific model. Proposed TTA: patient‐specific model with test‐time adaptation.

Furthermore, we evaluated the number of data fractions required to train a small sample set to achieve high accuracy. As shown in Figures 3 and 4, model performance gradually improved with an increasing number of applied fractions. For target volumes and OARs with well‐defined boundaries and minimal motion (e.g., certain anatomical structures), excellent training outcomes were achieved using only one or two fractions. However, for OARs such as the small intestine and rectum, which exhibit poorly defined boundaries on CBCT images and significant motion between treatment fractions, the number of training fractions must be increased progressively to gradually achieve satisfactory results. Figure 5 shows representative multi‐view images of the contours generated by the model in a test case. The proposed model was superior to the DIR method in terms of contour accuracy and smoothness.

**FIGURE 3:**
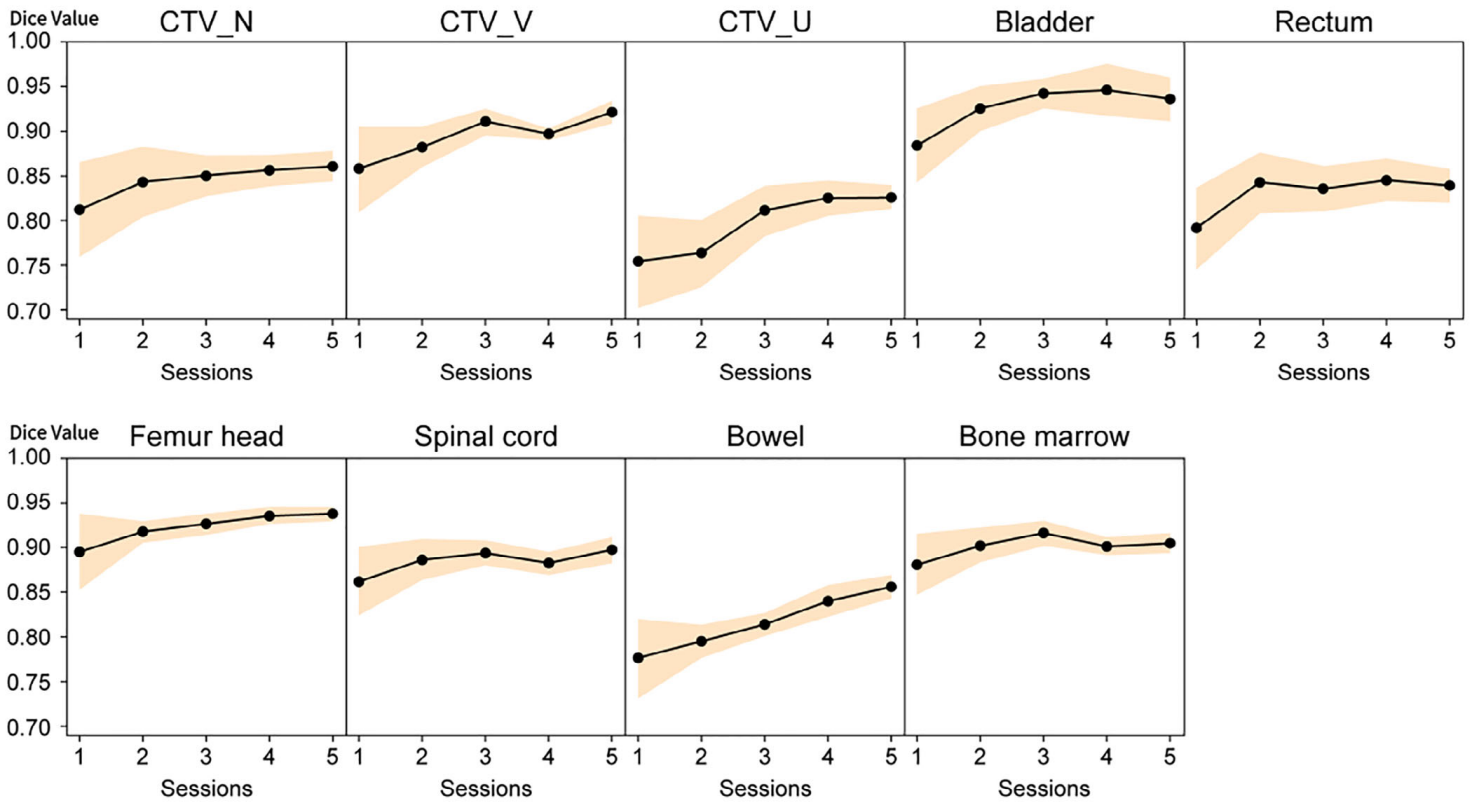
Dice similarity coefficient of the proposed TTA method according to the number of fractions.

**FIGURE 4:**
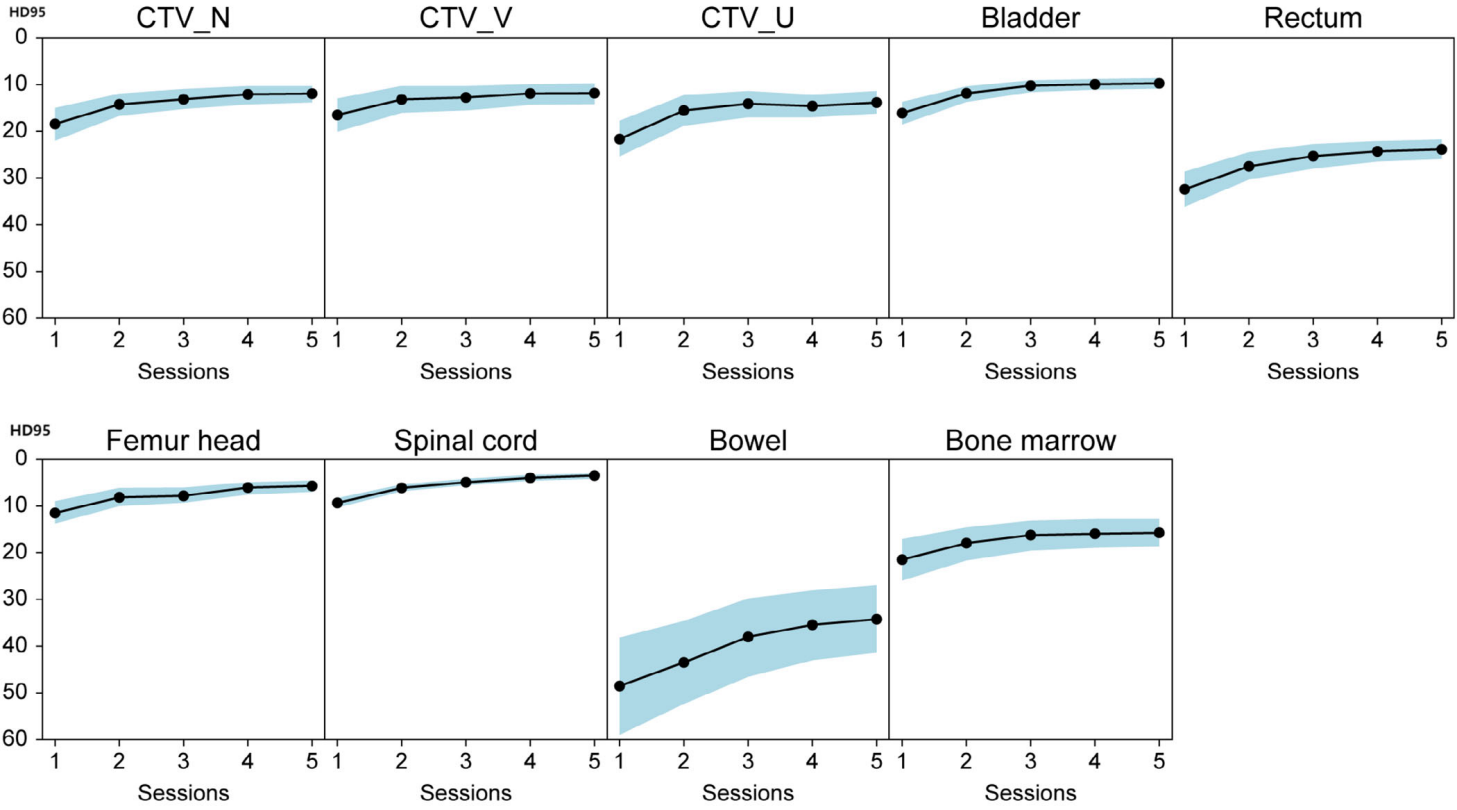
95% Hausdorff Distance of the proposed TTA method according to the number of fractions.

**FIGURE 5:**
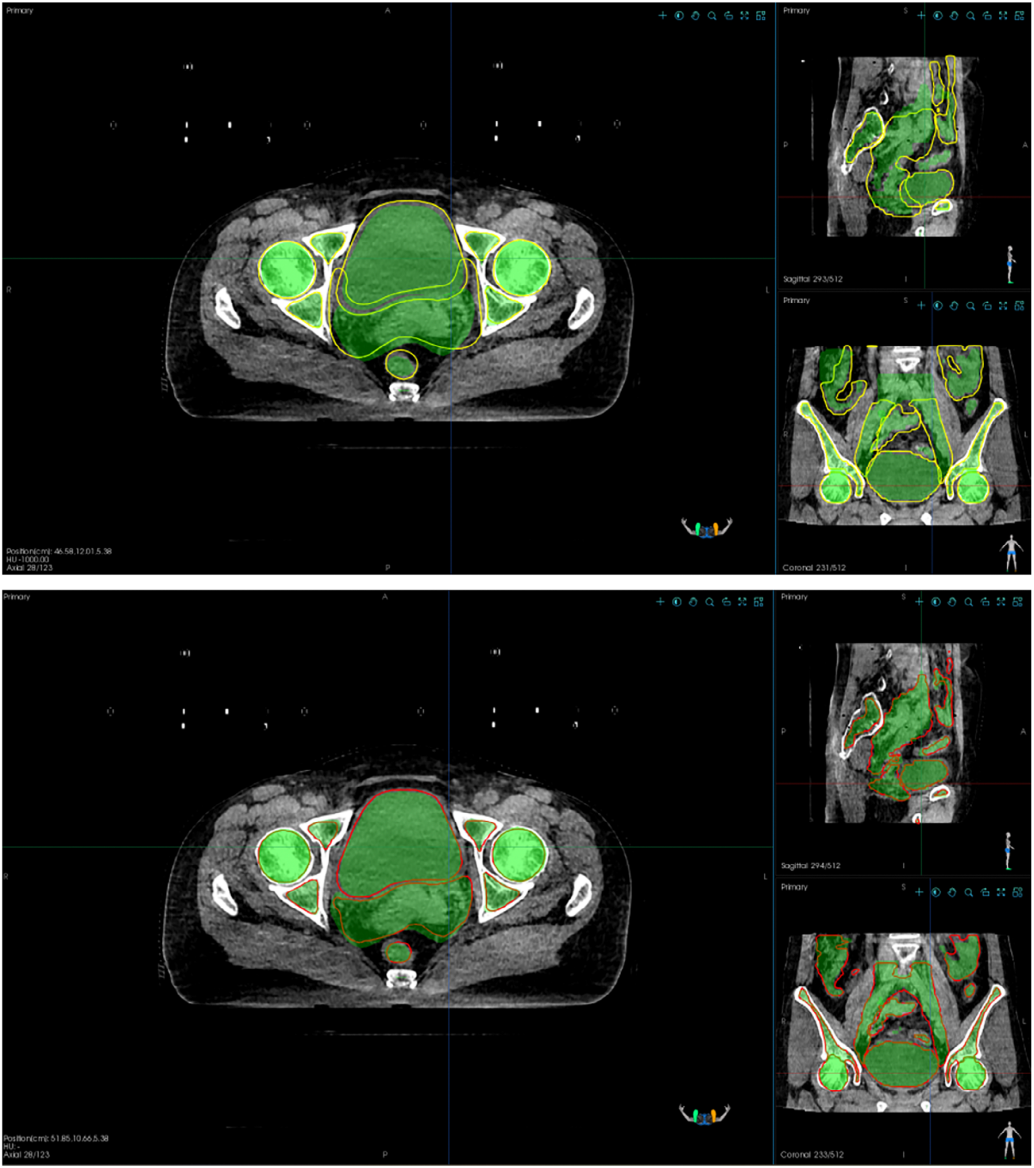
Example of segmentation performance between the proposed model‐based approach and deformation‐based methods. Green: manual segmentation; yellow: deformation‐based segmentation; red: proposed TTA segmentation.

## 4 DISCUSSION

In this study, we propose a practical solution to enhance the efficiency of the contouring workflow in CBCT‐guided adaptive radiotherapy for cervical cancer through few‐shot learning‐based auto‐segmentation. Our patient‐specific auto‐segmentation method employs a deep learning model that is updated daily and accounts for anatomical variations during treatment.

Previous automated contouring studies predominantly rely on large prior models.^18^, ^19^, ^20^ Although these approaches perform well in routine cases, they exhibit significant variability in CBCT‐based online adaptive radiotherapy.^21^, ^22^, ^23^, ^24^ First, current clinical practice indicates that online adaptive radiotherapy is particularly suitable for patients with substantial daily target volume variations, many of whom present with rare diseases, recurrent tumors, or metastases. These complex cases exhibit intricate spatial relationships between targets and OARs, where minor deformations can critically affect treatment quality. Unlike standard treatment cases (e.g., nasopharyngeal or rectal cancers), which benefit from abundant labeled data for model training, these special cases suffer from data scarcity. Our few‐shot learning approach effectively addressed this challenge while maintaining clinical workflow compatibility.^25^, ^26^According to previous studies,^1^, ^2^, ^3^ the Varian ETHOS built‐in AI auto‐contouring system has demonstrated good accuracy across various anatomical sites. For organs‐at‐risk (OAR), structures with clear boundaries and larger volumes achieve higher Dice scores: bladder (∼0.94) and femoral heads (∼0.95). In contrast, smaller organs with ambiguous boundaries show poorer performance: thyroid (∼0.63) and duodenum (∼0.32). For target volumes, although DIR with structure‐guided deformation is employed, accuracy remains challenging. Previous studies reported that the current ETHOS target generation achieves a mean Dice coefficient of 0.93 ± 0.05 for GTV, while the median Dice value for pelvic nodal CTV ranges from 0.64 to 0.75 depending on the delineation method. The CTV generation results are similar to those reported using the deformable registration method in our study, highlighting the limitations of this approach.^27^, ^28^, ^29^

Second, CBCT image quality inherently differs from that of diagnostic CT and MRI because of modality‐specific limitations in noise and artifacts. Although previous studies have reported few‐shot models for gross tumor volume delineation on MRI (achieving mean DSC values > 0.9 through superior soft‐tissue contrast), our work represents the first attempt at few‐shot CTV delineation on CBCT. Our results demonstrate differential learning requirements: OARs with clear boundaries and minimal deformation achieve satisfactory segmentation with one or two fractions, whereas organs with ambiguous boundaries or significant intra‐fractional motion require four or five fractions for optimal performance.

Unlike generic TTA methods (e.g., TENT, SAR, or CoTTA) that adapt a fixed population‐level model to new domains, our approach personalizes the model per patient from the outset and leverages temporal consistency across treatment fractions—a strategy uniquely suited to the longitudinal nature of fractionated radiotherapy. The use of rigid registration ensures stable alignment of skeletal landmarks (e.g., pelvic bones), providing a reliable spatial reference frame. Soft‐tissue motion is not enforced by an external deformation field but is instead learned implicitly through the adaptive segmentation process, making our approach more robust to DIR failures.

The improved segmentation accuracy translates into a time savings of 4–6 min per fraction. This temporal efficiency is crucial for pelvic adaptive radiotherapy, as a prolonged online workflow duration exacerbates intra‐fractional variations. Unlike random inter‐fractional errors that average out over treatment, systematic intra‐fractional errors (caused by bladder filling or bowel movement) may accumulate directional displacements, increasing the risks of target underdosage and OAR toxicity. Our method enhanced the overall treatment efficiency by approximately 20%, The trade‐off between computational time and manual contouring time savings is a critical consideration for the clinical feasibility of Online Adaptive Radiotherapy (Online ART). Specifically, 500 iterations of TTA were implemented on an NVIDIA A100 GPU with optimized model architecture and batch processing, yielding an average adaptation time of 28.6 ± 4.2 s per fraction; this computational overhead is negligible compared to the manual contouring time savings (230–250 s per fraction) and does not negate the net workflow improvement. The efficiency of TTA iterations is attributed to two key optimizations: (1) the lightweight architecture of the patient‐specific model, which incorporates residual modules and a BYOL‐pre‐trained efficient encoder to reduce redundant computations, and (2) the use of rigid registration (instead of DIR) for spatial alignment, which avoids the high computational cost of DIR while maintaining stability through skeletal landmark alignment. Potentially reducing planning target volume margins and toxicity risks after clinical validation. Moreover, the improved temporal consistency facilitates a more accurate assessment of intra‐fractional variations for an optimized margin design.

Nevertheless, we acknowledge a few limitations of our study. All segmentation tasks in this study were based on few‐shot learning. For structures requiring 4–5 fractions to achieve clinically acceptable DSC indices, subsequent research could adopt differentiated strategies: (1) for well‐performing OARs or targets, continue using few‐shot models; (2) for suboptimal OAR segmentation, integrate large‐scale OAR segmentation models; and (3) for target structures, leverage pre‐treatment CT‐based contours and deformable registration techniques to rapidly enhance the initial segmentation accuracy. This multi‐strategy approach can better support clinical implementation while maintaining workflow efficiency. All data were retrospectively collected from a single academic center, which may limit generalizability. However, our cohort includes 20 patients with diverse treatment scenarios (postoperative vs. definitive radiotherapy) and 530 daily CBCT scans, capturing substantial intra‐patient anatomical variability. Prospective multi‐center validation is currently underway in collaboration with two additional institutions. While our study focused on geometric and workflow outcomes, future work will incorporate dosimetric analysis to quantify how TTA‐driven contours affect target coverage and OAR sparing compared to DIR or manual contours.

Another limitation of our contouring time analysis is its retrospective and offline nature. Radiation oncologists performed their evaluations knowing that their edits would not directly impact patient care, which may have resulted in a different (potentially less stringent or more relaxed) review process compared to a real oART session under time pressure. Consequently, the reported time savings of approximately 4–6 min per fraction should be interpreted as an upper‐bound estimate of efficiency gain achievable in an ideal, non‐clinical‐pressure setting. The actual time benefit in a live clinical workflow may vary and warrants prospective investigation.

Our study utilized CBCT images reconstructed with the standard iterative algorithm available on the Ethos platform at the time of data collection (October 2022–March 2023). Subsequent advancements, such as the HyperSight reconstruction technique, have demonstrably improved CBCT soft‐tissue contrast and image quality. While our method proved effective on the image quality of its era, its performance and the magnitude of its benefits may be further enhanced—or require re‐evaluation—when applied to the superior image quality provided by HyperSight. This represents a temporal limitation of our current dataset but also establishes a foundation for future validation on next‐generation imaging.

## 5 CONCLUSION

We present the first retrospective demonstration of a TTA‐driven patient‐specific segmentation approach for online adaptive radiotherapy using pelvic CBCT images. The proposed methodology effectively addresses several critical limitations of CBCT‐based deep learning segmentation, including image noise, artifacts, CT number instability, and data scarcity. Our results demonstrate that this approach has the potential to streamline the clinical workflow for CBCT‐guided online adaptive radiotherapy by improving operational efficiency, while potentially providing clinical benefits by mitigating intra‐fractional variations. Future investigations will focus on enhancing the overall segmentation accuracy by incorporating diverse contouring strategies and methodological refinements.

## AUTHOR CONTRIBUTIONS

**Zhiqun Wang**: Conceptualization; methodology; investigation; data curation; writing — original draft. **Wei Zhang**: Conceptualization; methodology; investigation; data curation; writing — original draft. **Xiangyin Meng**: Validation; formal analysis; writing — review & editing. **Yongguang Liang**: Investigation; resources; writing — review & editing. **Tingting Dong**: Investigation; visualization; writing — review & editing. **Jie Zhang**: Data curation; formal analysis; writing — review & editing. **Nan Liu**: Resources; data curation; writing — review & editing. **Lang Yu**: Investigation; validation; writing — review & editing. **Bo Yang**: Supervision; project administration; funding acquisition; writing review & editing. **Qichao Zhou**: Supervision; project administration; funding acquisition; writing review & editing. **Jie Qiu**: Supervision; project administration; conceptualization; funding acquisition; writing — review & editing.

## CONFLICT OF INTEREST STATEMENT

The authors declare no conflicts of interest.

## ETHICS STATEMENT

This study was reviewed and approved by the Ethics Review Committee of Peking Union Medical College Hospital, Chinese Academy of Medical Sciences (Approval No.: I‐24PJ0173).

## Data Availability

The data that support the findings of this study are available on request from the corresponding author. The data are not publicly available due to privacy and ethical restrictions.
